# Supplementary material for: A Bayesian approach to combining multiple information sources: Estimating and forecasting childhood obesity in Thailand
Source: PLoS One. 2022 Jan 21;17(1):e0262047. doi: 10.1371/journal.pone.0262047 (PMC8782526; doi:10.1371/journal.pone.0262047)
Supplement: S1 File — (PDF) [file pone.0262047.s001.pdf]

Supporting information for 'A Bayesian approach to combining multiple information sources: Estimating and forecasting childhood obesity in Thailand'

Additional direct estimates of obesity prevalence

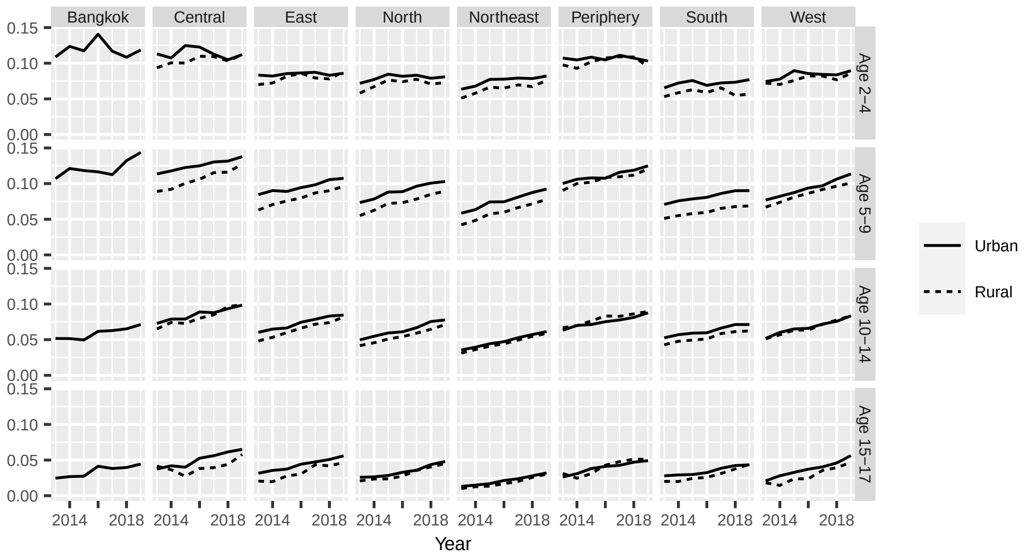

Fig 1. Direct estimates of obesity prevalence by age, region, and urban-rural residence for males, based on schools data

Converting NHES data into effective counts

Here we describe how we convert the raw National Health Examination Survey data into effective counts.

Let o_i be 1 if the ith respondent in an NHES sample is obese, and 0 otherwise; let w_i be the survey weight for that respondent; let η_{as} denote the set of all respondents in

age group a and sex s; and let $\tilde{n}_{as}$ be the number of respondents. We calculate

$$p_{as} = \frac{\sum_{i \in \eta_{as}} w_i o_i}{\sum_{i \in \eta_{as}} w_i},$$

the obesity prevalence within that combination of age, sex, and year, estimated using survey weights. We also calculate the variance in weights

$$V_{as} = \frac{\sum_{i \in \eta_{as}} (w_i - \bar{w}_{as})^2}{\tilde{n}_{as} - 1},$$

and define design effect

$$d_{as} = 1 + \frac{V_{as}}{\bar{w}_{as}^2}.$$

The value d_{as} measures the extent to which sampling in age-sex group as departs from simple random sampling. The higher the value of d_{as}, the greater the departure. Let d^* denote the median value for d_{as}. We define effective sample size as $n_{as} = \tilde{n}_{as}/d^*$ and define the effective number of obese respondents as $y_{as} = p_{as} n_{as}$. Before applying our model, we round n_{as} and y_{as} to obtain whole numbers.

This procedure is identical to that in [1], except that we use the median design effect, rather than the mean, on the grounds that the median is more robust to outliers.

Dropping the age-specific bias term from the data model for schools

Fig 2 below shows national estimates and forecasts when the data model for schools does not have an age-specific bias term. Fig 3b in the main text shows the equivalent estimates and forecasts when the data model for schools does have an age-specific bias term. Without the bias term, there is one-off change in apparent prevalences for children aged 2–4 when moving from the period covered by the NHES and HDTC data to the period covered by the schools data. The widths of the credible intervals are also reduced. The one-off change appears implausible. Given that there is uncertainty about age-specific biases, the wider credible intervals of the main model are, we believe, more appropriate.

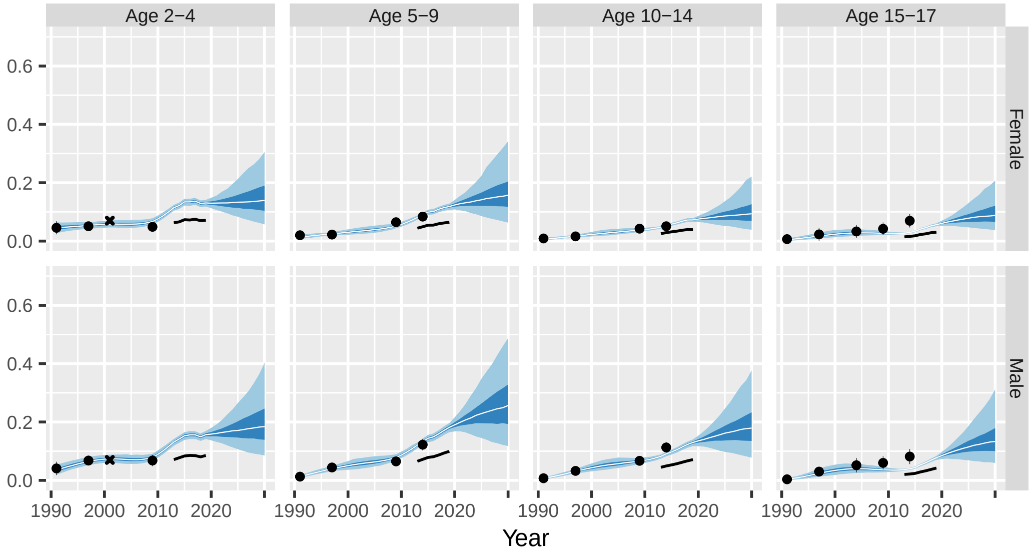

Fig 2. Estimates and forecast of obesity prevalence using a data model for schools that does not include an age-specific bias term

Converting WHO obesity estimates into effective counts

Our procedure for converting WHO obesity estimates into effective counts is a modified version of the procedure for turning NHES data into effective counts.

Let p_{cst} be the estimated prevalence for sex s in country c in year t. Let w_{cst} be the width of the associated 95% confidence interval. The width of a 95% confidence interval should equal approximately 4 standard deviations. If we treat p_{cst} as being derived from the effective number of obese respondents divided by the effective number of respondents n'_{cst}, then one standard deviation equals $\sqrt{n'_{cst}p_{cst}(1-p_{cwt})}$. We solve the equation

$$w_{cst} = 4\sqrt{n'_{cst}p_{cst}(1-p_{cwt})} \tag{1}$$

to obtain a value for n_{cst}. Individual values of n'_{cst} can be very noisy, so we set n^{W}_{cwt} equal to the median of n'_{cst} across s and t. We then set $y^{\mathrm{W}}_{cst} = p_{cst}n^{\mathrm{W}}_{cwt}$.

Deriving prior distributions from WHO data

Having fitted our model to the WHO data we obtain a sample $\tau_\beta^{\mathrm{WHO}(k)}, k = 1, \cdots, K$ from the posterior distribution for $\tau_\beta^{\mathrm{WHO}}$. We calculate $S^2 = \sum_{k=1}^{K} (\tau_\beta^{\mathrm{WHO}(k)})^2$, and use $N(0, S^2)$ as our initial prior for τ_β in (4) in the main model. We obtain initial priors for τ_α and τ_δ in the same way.

The fitted model for the WHO data also yields sample $\phi^{\mathrm{WHO}(k)}$ from the posterior distribution for ϕ^{WHO}. We set $\tilde{\phi}^{\mathrm{WHO}} = \frac{\phi^{\mathrm{WHO}(k)-0.8}}{1-0.8}$, and calculate $m = \sum_{k=1}^{K} \tilde{\phi}^{\mathrm{WHO}(k)}$ and $V = \sum_{k=1}^{K} (\tilde{\phi}^{\mathrm{WHO}(k)} - m)^2/K$. We set $a = m\left(\frac{m(1-m)}{v} - 1\right)$ and $b = (1-m)a$, and use $\mathrm{Beta}(a, b)$ as our initial prior for ϕ in the main model.

As discussed in the main text, we suspect that our initial posterior distributions for τ_β, τ_α, τ_δ, and ϕ may understate true year-to-year variability. We therefore construct modified versions of the priors that allow for greater variability. Let k be a multiplier, which in practice we set to 2 and 4. Our modified version of the prior for τ_β is simply $N(0, (kS)^2)$, and similarly for τ_α and τ_δ. To obtain the modified version of the prior for ϕ, we multiply V by k.

Additional results from Models 4 and 5

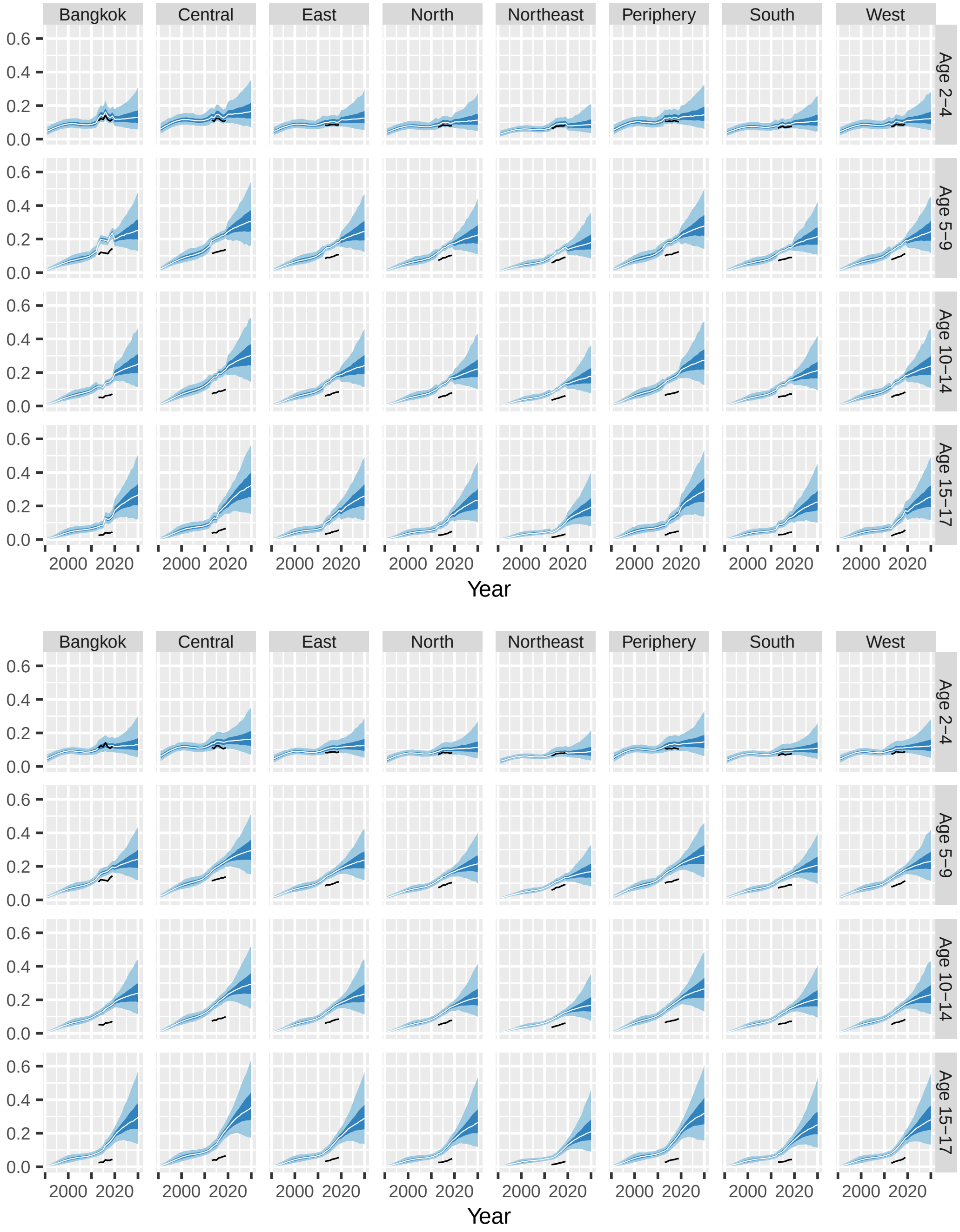

Fig 3. Estimates and forecasts of obesity prevalence for males in urban areas, from Model 4 (top) and Model 5 (bottom)

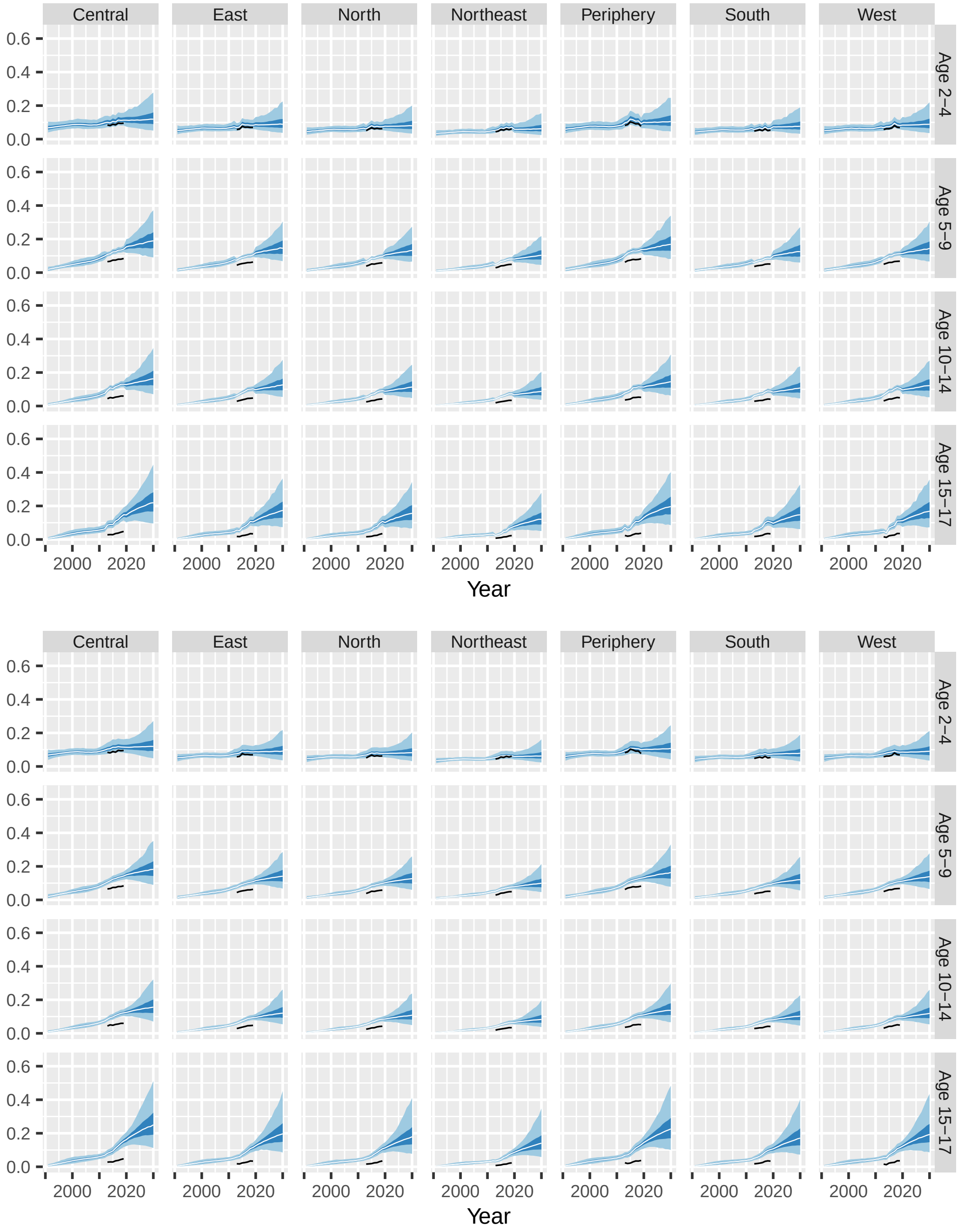

Fig 4. Estimates and forecasts of obesity prevalence for females in rural areas, from Model 4 (top) and Model 5 (bottom)

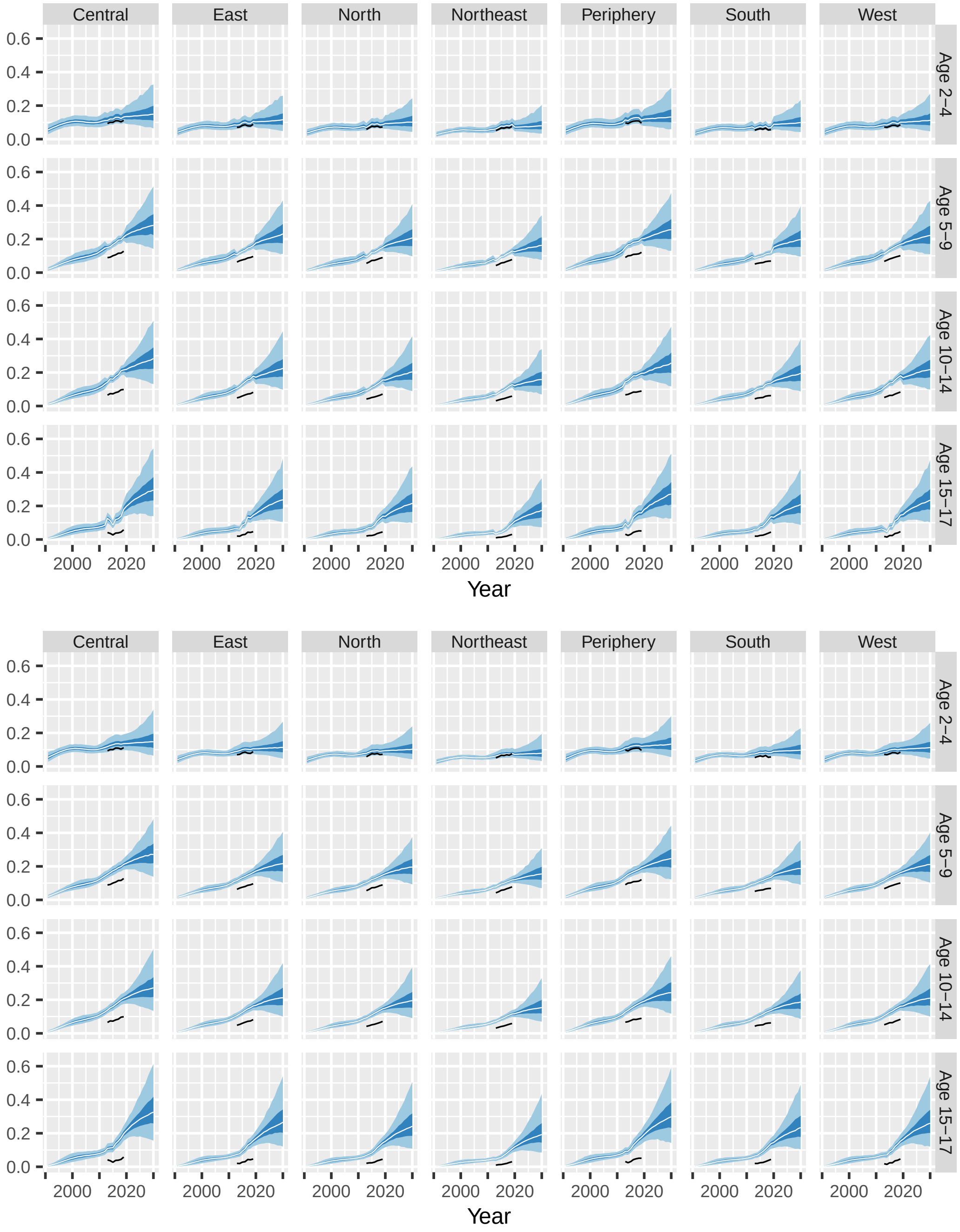

Fig 5. Estimates and forecasts of obesity prevalence for males in rural areas, from Model 4 (top) and Model 5 (bottom)

References

1.

Ghitza Y, Gelman A. Deep interactions with MRP: Election turnout and voting patterns among small electoral subgroups. American Journal of Political Science. 2013;57: 762–776.
