# Supplementary material for: A Bayesian approach to combining multiple information sources: Estimating and forecasting childhood obesity in Thailand
Source: PLoS One. 2022 Jan 21;17(1):e0262047. doi: 10.1371/journal.pone.0262047 (PMC8782526; doi:10.1371/journal.pone.0262047)
Supplement: Attachment — Submitted filename: Response to Reviewers.pdf [file pone.0262047.s002.pdf]

PONE-D-21-23949
A Bayesian approach to combining multiple information sources: Estimating and forecasting childhood obesity in Thailand
PLOS ONE

Reviewer's comments	Responses
Journal Requirements:<br>When submitting your revision, we need you to address these additional requirements.<br>1. Please ensure that your manuscript meets PLOS ONE's style requirements, including those for file naming. The PLOS ONE style templates can be found at https://journals.plos.org/plosone/s/file?id=wjVg/PLOSOne_formatting_sample_main_body.pdf and https://journals.plos.org/plosone/s/file?id=ba62/PLOSOne_formatting_sample_title_authors_affiliations.pdf	We have reformatted the manuscript to meet PLOS ONE's style requirements, including figure captions.
2. We note that you have stated that you will provide repository information for your data at acceptance. Should your manuscript be accepted for publication, we will hold it until you provide the relevant accession numbers or DOIs necessary to access your data. If you wish to make changes to your Data Availability statement, please describe these changes in your cover letter and we will update your Data Availability statement to reflect the information you provide.	Data and code for the WHO-based prior distributions are at https://github.com/johnrbryant/bayescombwho and data and code for the rest of the paper are at https://github.com/johnrbryant/bayescomb
3. Please include your full ethics statement in the 'Methods' section of your manuscript file. In your statement, please include the full name of the IRB or ethics committee who approved or waived your study, as well as whether or not you obtained informed written or verbal consent. If consent was waived for your study, please include this information in your statement as well.	This study does not involve primary data and used only secondary datasets from various sources. The ethics statement (Code of Exemption) is included in the Data section.<br><br>Ethics statement<br>This study was approved by the Institute for Population and Social Research 107 Institutional Review Board (IPSR-IRB), at Mahidol University, Thailand (COE. 108 No. 2019/07-278).

4. Please include captions for your Supporting Information files at the end of your manuscript, and update any in-text citations to match accordingly. Please see our Supporting Information guidelines for more information: http://journals.plos.org/plosone/s/supporting-information.	We included captions in the Supporting Information file, and have updated all in-text citations.
5. Please review your reference list to ensure that it is complete and correct. If you have cited papers that have been retracted, please include the rationale for doing so in the manuscript text, or remove these references and replace them with relevant current references. Any changes to the reference list should be mentioned in the rebuttal letter that accompanies your revised manuscript. If you need to cite a retracted article, indicate the article's retracted status in the References list and also include a citation and full reference for the retraction notice.	We have reviewed all references to ensure that they have not been retracted. The reference format was checked and completed.
Reviewers' comments:	
Reviewer's Responses to Questions<br>**Comments to the Author**<br><br>1. Is the manuscript technically sound, and do the data support the conclusions?<br><br>The manuscript must describe a technically sound piece of scientific research with data that supports the conclusions. Experiments must have been conducted rigorously, with appropriate controls, replication, and sample sizes. The conclusions must be drawn appropriately based on the data presented.<br>Reviewer #1: Yes<br>Reviewer #3: Yes	Thank you
2. Has the statistical analysis been performed appropriately and rigorously?<br>Reviewer #1: Yes<br>Reviewer #3: Yes	Thank you

3. Have the authors made all data underlying the findings in their manuscript fully available?<br><br>The PLOS Data policy requires authors to make all data underlying the findings described in their manuscript fully available without restriction, with rare exception (please refer to the Data Availability Statement in the manuscript PDF file). The data should be provided as part of the manuscript or its supporting information, or deposited to a public repository. For example, in addition to summary statistics, the data points behind means, medians and variance measures should be available. If there are restrictions on publicly sharing data—e.g. participant privacy or use of data from a third party—those must be specified.<br>Reviewer #1: Yes<br>Reviewer #3: Yes	Thank you
4. Is the manuscript presented in an intelligible fashion and written in standard English?<br><br>PLOS ONE does not copyedit accepted manuscripts, so the language in submitted articles must be clear, correct, and unambiguous. Any typographical or grammatical errors should be corrected at revision, so please note any specific errors here.<br>Reviewer #1: Yes<br>Reviewer #3: Yes	Thank you
5. Review Comments to the Author<br><br>Please use the space provided to explain your answers to the questions above. You may also include additional comments for the author, including concerns about dual publication, research ethics, or publication ethics. (Please upload your review as an attachment if it exceeds 20,000 characters)	The explanation responds to each comments shown above.
Reviewer #1:	

I think that the paper is well written and the gradual build-up, from simple to more complex models, makes it easy to follow authors' presentation and to see how different information that are combined from various sources in ultimately constructing a model to analyze and predict children obesity in Thailand. I am listing here a few concerns that I hope perhaps the authors can clarify in the paper in order for a better understanding from the users.	Thank you
(1) after the first model, information from HDTC and Schools data are used to strengthen inference. Instead of using observed data, you estimated the truth population total (n's) and true obese children (y's). They become the data models. Once you have three models, you combined them in a single analysis. Can you be more specific on how this combined analysis is carried out? My confusion, perhaps a misunderstanding, is that if you estimate three true population totals (n^TRUE's) and three y^TRUE, and if you naively combine them, estimation would shrink the standard errors because you over count the population total: p(1-p)/n if n gets to be artificially large (3xn^TURE).	This is an interesting point, and we suspect that the same question may occur to other readers. We have added the following paragraph to methodological description of the first model (page 13, line 322):<br><br>"The likelihood from combining the three datasets has the form<br><br>p(data|true obesity counts) = p(NHES data|true obesity counts) × p(HDTC data|true obesity counts)<br>× p(Schools data|true obesity counts).<br><br>The (unobserved) true obesity counts appear multiple times in the likelihood. The repetition does not, however, cause any problems. It is analogous to having the same regression coefficients occur multiple times in the likelihood for a regression model."

(2) model 1, equation 4-6, page 8. That time-dependent 'damped linear trend' model has its advantages in offering a way to model how the time effects change. But it seems to give you some trouble, and estimation is sensitive to the variance parameters, e.g. tao_delta^2, and later you pointed out that, when more information is used, you ended up gaining a better estimate of these parameters, which help in stabilizing the estimates, etc. I feel that there are other practical ways of modeling time-varying beta_t coefficients jointly, without having to resort to estimating the random drifts. For example, multivariate normal prior with certain temporal covariance type. Those probably can also be more computationally stable, and less sensitive to the choices of the prior. Have you considered using alternatives?	It is true that a less flexible prior for the time effects would be easier to estimate, especially given our limited number of observations. However, it seems that we need all of the features of the damped linear trend, in order to capture the main features of the data. For instance, it would be much simpler if we could assume that rates of change were constant over time, but the evidence suggest that rates of change for teenagers have in fact increased since 2010. The software we are using does not give a wide choice of priors for time effects. However, we suspect that other priors with similar levels of flexibility would lead to similar difficulties.<br><br>We have added the following paragraph to our discussion of the damped linear trend prior (page 9, line 202-207):<br><br>“As we discuss below, the flexibility of the damped linear trend prior makes it challenging to fit. However, less flexible versions of the prior could potentially miss important features of the data. It is, for instance, tempting to assume that, within each combination of age and sex, rates of change are constant over time. Doing so would, however, reduce our ability to detect turning points, and could produce forecasts that were inappropriately confident.”

(3) Why some data models you use normal likelihood (e.g. Eq 9), others you take log transformation (Eq 10)?<br><br>(3a) Your data models depend on externally-estimated parameter, such as kappa_ast in model of y^EstN_ast. Can you provide a bit more explanations, at least intuitively, why that is a reasonable idea?	We have completely rewritten the paragraphs describing Eq 9. The new paragraphs are as follows (page 12, line 288-298):<br><br>"To construct our data model for the NHES, we rely on features of the design of the survey, which is a common strategy in Bayesian analyses of multiple data sources [8,15]. The design of the survey implies that the NHES estimates should be unbiased, and that errors in these estimates should be approximately normally distributed. Moreover, the standard deviations for these errors can be estimated through design-based methods<br>that exploit information about the survey including sample weights.<br><br>Our data model for the NHES is<br>yEstN ~N(yTrue,κ2) (9)<br>where the superscript 'EstN' denotes 'estimates derived from the NHES'. We set the κast equal to the standard deviations that the R package survey produces alongside the estimates yTrue. (Code for the design-based calculations s included in the repository https://github.com/johnrbryant/bayescomb.)<br>"<br><br>In addition, to motivate the use of log units rather than natural units in the schools data model, we have added the following sentence (page 13, line 310-311):<br><br>"The use of logs implies that the data model is expressed in terms of percentage errors, rather than absolute errors."
(3b) you indicate that, while principally it is possible to estimate all systematic differences in data model, you end up choosing only to include age-specific gamma_alpha, and explain that having too many such bias parameters would make the estimation more difficult. To me, that is an indicate that not enough	We have added a sensitivity test where, as suggested, we drop the gamma_alpha term. The results, shown in section "Dropping the age-specific bias term from the data model for schools" in the Supplementary Materials.The gamma_alpha term is in fact needed. In the main section of the paper we

information in the data can be used to estimate such bias parameters (theoretically, but practically need more data). This leads to a question that perhaps you can address in the paper: is it then even necessary to include the gamma_alpha parameter? Will your model fit be just as good if that parameter is not there? Maybe this amounts to doing some sensitivity analysis in comparing models with or without that parameter and see if any significant different conclusions can be observed.	write (Supplementary Material, page 2, line 18-28)<br><br>“Dropping the age-specific bias term from the data model for schools<br><br>Fig 2 below shows national estimates and forecasts when the data model for schools does not have an age-specific bias term. Fig 3b in the main text shows the equivalent estimates and forecasts when the data model for schools does have an age-specific bias term. Without the bias term, there is one-off change in apparent prevalences for children aged 2–4 when moving from the period covered by the NHES and HDTC data to the period covered by the schools data. The widths of the credible intervals are also reduced. The one-off change appears implausible. Given that there is uncertainty about age-specific biases, the wider credible intervals of the main model are, we believe, more appropriate.”
(4) minor point: page 3, line 61, what are bayescomb and bayescombwho?	These were supposed to be hyperlinks to GitHub repositories, but did not print correctly. We have replaced these links.
Reviewer #3:	

I thank the authors for investigating such an important subject; I have some minor comments:<br><br>- Method and Results should be written in separate sections.	We have added "Methods" and "Results" subheadings to the descriptions of Models 1, 2, 3, 4, 5.
- Line 53-55: the third extension is missing.	Thank you for alerting us to the missing third extension! We have renumbered the extensions.
- Line 173-180: Authors assigned Normal-prior to variables Sex and Age. Considering the positive nature of the indicator variable, why did not the authors use the half-normal prior for these variables?	We have added a sentence (page 7, line 169), "Transformation to the logit scale implies that values are no longer bounded by 0 and 1."<br><br>We illustrate the implications for the sex prior with the sentence (page 10, line 180) "This prior captures the idea that, on a logit scale, we might see female-male differences of -0.1 or 1.2, for instance, but not -10 or 120." (Previously we used the numbers 0.1 and 10, rather than -0.1 and -10.)
6. PLOS authors have the option to publish the peer review history of their article (what does this mean?). If published, this will include your full peer review and any attached files.<br><br>If you choose "no", your identity will remain anonymous but your review may still be made public.<br><br>**Do you want your identity to be public for this peer review?** For information about this choice, including consent withdrawal, please see our Privacy Policy.<br><br>Reviewer #1: No Reviewer #3: No	Sorry, we made a mistake. This should be revised to be "yes". All authors have agreed to reveal their identity including name, title, and workplace.
